# Incidental Discovery of a Right Atrial Diverticulum in an Adult Patient

**DOI:** 10.3390/diagnostics15162058

**Published:** 2025-08-16

**Authors:** Viviana Onofrei, Iuliana Rusu, Oana-Mădălina Manole

**Affiliations:** 1 Internal Medicine Department, “Grigore T. Popa” University of Medicine and Pharmacy, 700115 Iasi, Romaniamanole.oana-madalina@d.umfiasi.ro (O.-M.M.); 2 Cardiology Department, “Saint Spiridon” Emergency Clinical County Hospital, 700111 Iasi, Romania

**Keywords:** congenital malformation, right atrium diverticulum, heart failure

## Abstract

Background and Clinical Significance: Congenital malformations of the right atrium are rare. Their clinical presentation varies widely, from the absence of symptoms to sudden death, often being diagnosed incidentally by cardiac imaging. First described in 1955, the right atrial diverticulum is usually characterized as a pouch-like structure originating from the free atrial wall, or right atrial appendage. The prevalence of congenital malformations of the right atrium is unknown because few clinical cases have been reported. Associated complications include arrhythmias, pulmonary thromboembolism, progressive dilatation marked by a high risk of compression and rupture. In these cases, the optimal therapeutic approach is surgical resection. Case Presentation: We present the case of a 58-year-old, hypertensive female with a history of COVID-19 (Coronavirus Disease 2019), who was admitted for persistent dyspnea and chest pain. An electrocardiogram on arrival showed no arrhythmias or ischemic changes, and echocardiography revealed severe systolic dysfunction—a left ventricular ejection fraction (LVEF) of 20%, moderate mitral and tricuspid regurgitations, and a pericardial collection, adjacent to the right atrium, considered to be a localized pericardial effusion. Coronary angiography excluded ischemic etiology and a viral myocarditis was further suspected. Cardiac magnetic resonance imaging (IRM) showed a non-ischemic scar pattern in the interventricular septum, but also detected a well-defined large mass, which communicated with the right atrium through a 20 mm opening, suggestive of a right atrial diverticulum. Contrast echocardiography confirmed the communication between the cavity and the right atrium. A surgical resection of the large diverticulum was performed. Conclusions: The particularity of this case consists in the incidental identification of a rare cardiac malformation in an adult patient.

## 1. Introduction

Congenital malformations of the right atrium are rare and are represented by congenital right atrium dilatation, single or multiple diverticula, and congenital aneurysms [1]. Idiopathic dilatation of the right atrium was first described in 1955 by Bailey et al. [2], and it is the most frequently reported form of idiopathic dilation in the existing literature. The incidence of each type of malformation is unknown due to underdiagnosis and the lack of clear distinction between the terms. It may occur sporadically, but familial aggregation has also been described [1]. It may be solitary or accompanied by other congenital malformations such as hypertrophic cardiomyopathy [3], and atrial or ventricular septal defects [4,5].

The right atrial diverticulum is a saccular outpouching that originates from the right atrial wall [6]. This has been identified in both children [5,7,8,9] and adult patients [10,11] as a single malformation [12] or multiple [13] malformations. The largest reported right atrial diverticulum was 20 × 16 cm [14]. The anatomical location of right atrial diverticula can be variable. The reported diverticula were placed posterior to the right atrium, retrocardiac (secondary to incomplete regression of the Cuvier ductus), or anterior to the right atrium with an appendicular origin [5,7,15,16].

Histologically, the diverticular wall consists of fibrous tissue and endothelium [12], often without a muscular layer or with only rare smooth muscle fibers [16].

Clinical presentation is variable, ranging from incidental diagnosis due to signs of compression (chest discomfort, dyspnea, turgescent jugular, hepatomegaly, peripheral edema) and palpitations caused by supraventricular refractory arrhythmias or complications (intracavitary thrombosis, pulmonary thromboembolism, rupture) [13,17,18]. The fibrous connective tissue, usually located between the right atrial diverticulum and the right atrium, acts as a substrate for arrhythmias [6,11].

In asymptomatic patients, the diagnosis starts from routine cardiac evaluation or cardiomegaly detection on chest X-rays [18], and is confirmed by specific cardiac imaging tests (transthoracic echocardiography, transesophageal echocardiography, coronary computed tomography angiography, magnetic resonance imaging) [4,11,16].

Treatment may be conservative in asymptomatic patients, and is also preferred when safe surgical resection of the right atrial diverticulum cannot be performed. It includes anticoagulants to avoid intracavitary thrombus formation [13]. Surgical resection is recommended for incidental large diverticula and symptomatic, complicated cases [17]. Radiofrequency ablation of tachyarrhythmias is usually performed after the surgical resection. For small diverticula, this can be performed before the surgical resection. Radiofrequency ablation of the epicardial right-sided accessory pathways is challenging and difficult, requiring complex and repeated procedures [11].

We summarized the case of a female, hypertensive patient, who presented for a two -year persistent dyspnea following a viral infection, in whom cardiac MRI confirmed myocarditis, but also showed a well-defined intrapericardial mass, communicating with the right atrium.

## 2. Case Presentation

We present the case of a 58-year-old woman with no significant past medical history, who was diagnosed with COVID-19 (Coronavirus Disease 2019) infection in 2021. The diagnosis was made by antibody testing following the onset of new shortness of breath and fatigue. The patient was not hospitalized and did not receive specific treatment. The shortness of breath persisted, and the patient presented to a general practitioner two years after the initial COVID-19 infection. Clinical examination and laboratory tests at that time excluded an active infection. The patient was diagnosed with arterial hypertension due to elevated blood pressure values and a treatment consisting of an angiotensin-converting enzyme inhibitor (perindopril 5 mg daily), a thiazide-like diuretic (indapamide 1.5 mg daily), and a beta-blocker (metoprolol 25 mg daily) was started. The patient also reported a family history of arterial hypertension. The patient adhered to the treatment, and home blood pressure was subsequently well controlled. Despite the adequate treatment, dyspnea worsened, prompting referral to the cardiology department.

**Figure 1:**
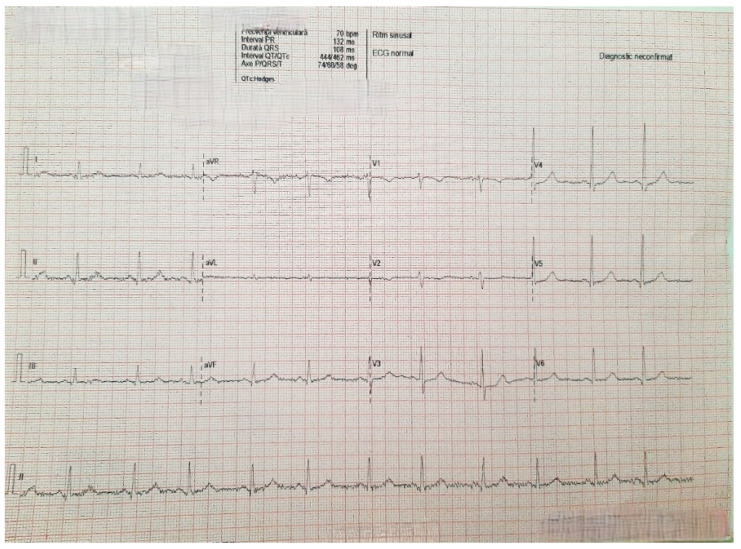
Electrocardiogram on admission showed sinus rhythm 75/min, QRS axis + 45 degrees, QRS with normal morphology.

Upon admission, we evaluated an overweight patient (a body mass index (BMI) of 28 kg/m^2^, an abdominal circumference of 90 cm, a height of 165 cm, and a weight of 76 kg). No other significant pathological findings were noted. The patient’s vital signs, including supine blood pressure (140/80 mmHg), standing blood pressure (145/80 mmHg), heart rate (80/min), and oxygen saturation on room air (98%), as well as the conventional electrocardiogram, were within normal limits (Figure 1).

Laboratory tests on admission revealed an elevated N-terminal pro–B-type natriuretic peptide (NT-proBNP) level of 2869 pg/mL, supporting the cardiac origin of the dyspnea, along with evidence of dyslipidemia (Table 1).

A chest X-ray demonstrated symmetrical lung fields without infiltrates or masses, clear cardiac contours, and a cardiothoracic ratio of 0.5. Transthoracic echocardiography revealed a non-dilated left ventricle without evidence of left ventricular hypertrophy, stage II diastolic dysfunction, and diffuse hypokinesis, more pronounced at the interventricular septum, with a volumetrically estimated left ventricular ejection fraction of 20%. The atria and right ventricle were of normal size, with a tricuspid annular plane systolic excursion (TAPSE) of 20 mm. Moderate pulmonary hypertension, as well as moderate mitral and tricuspid regurgitation, were detected. Pericardial fluid measuring 20 mm was noted lateral to the right atrium, interpreted as a localized pericardial effusion. Echocardiographic parameters are summarized in Table 2.

**Figure 2:**
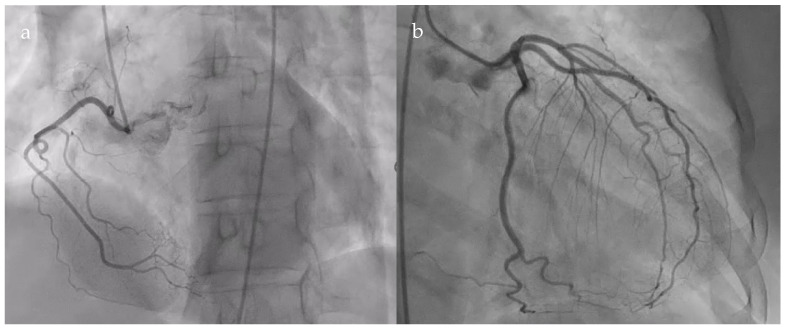
Normal coronary angiogram. (**a**). Right coronary artery; (**b**). Left main coronary artery, anterior descending artery, circumflex artery, and collateral branches.

The patient underwent coronary angiography to exclude an ischemic cause of the dyspnea. Coronarography showed normal epicardial coronary arteries (Figure 2).

Heart failure therapy was subsequently optimized by initiating a sodium–glucose cotransporter-2 inhibitor (dapagliflozin 10 mg once daily), an angiotensin receptor–neprilysin inhibitor (sacubitril/valsartan 24/26 mg twice daily), and a mineralocorticoid receptor antagonist (spironolactone 25 mg once daily), in addition to ongoing beta-blocker therapy (carvedilol 3.125 mg twice daily). The patient was also started on lipid-lowering therapy with a fixed-dose combination of rosuvastatin/ezetimibe (40/10 mg once daily).

The clinical course was interpreted as consistent with post-viral myocarditis secondary to COVID-19 infection. Given that the patient was not evaluated by a cardiologist during the acute viral infection, the assessment of the etiology of her dyspnea was delayed. Consequently, the patient did not receive targeted therapy, allowing the myocarditis to progress to a chronic stage.

The patient was discharged with a final diagnosis of hypokinetic, non-dilated cardiomyopathy secondary to post-viral myocarditis, localized pericardial effusion, arterial hypertension, and heart failure with reduced ejection fraction. Cardiac magnetic resonance imaging (MRI) was recommended to further evaluate the presence and extent of myocarditis.

Two months post-discharge, cardiac MRI demonstrated a notable improvement in left ventricular systolic function, with the ejection fraction increasing to 42%, though hypokinesia persisted in the interventricular septum. Early T1 mapping demonstrated a mildly increased signal in the interventricular septum, while T2-weighted imaging was unremarkable. Late gadolinium enhancement (LGE) sequences revealed a non-ischemic, mid-myocardial pattern of contrast uptake involving the interventricular septum and right ventricular insertion points, findings consistent with chronic myocarditis.

**Figure 3:**
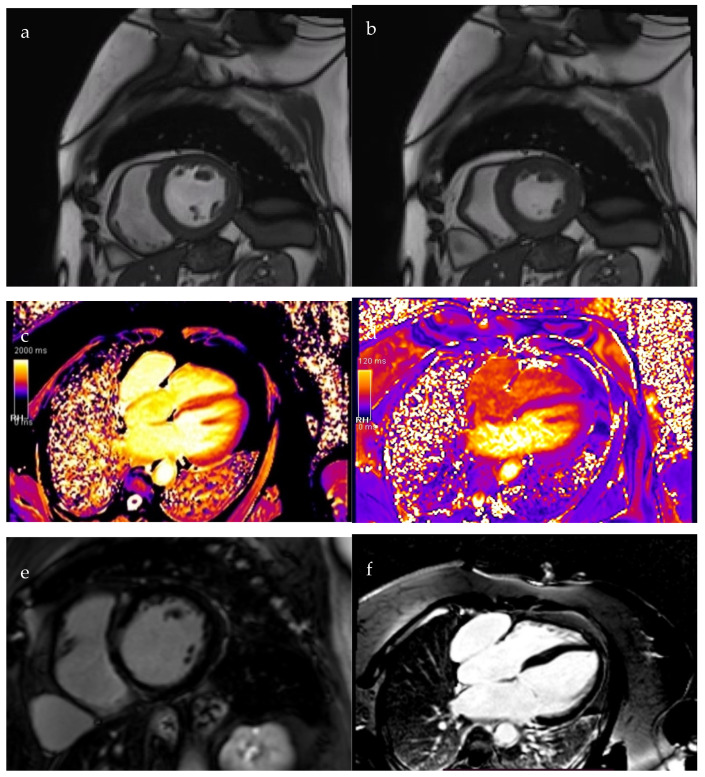

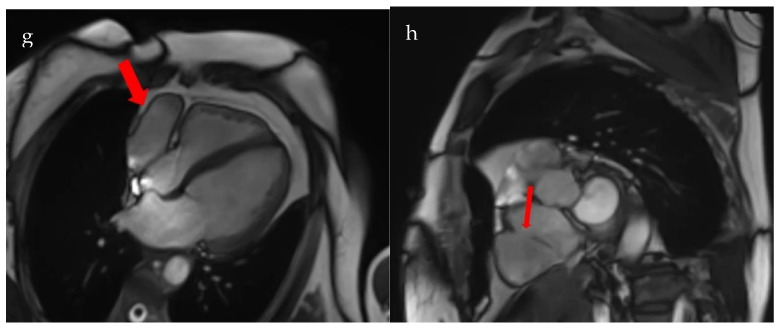
Cardiac MRI. (**a**). Diastolic view; (**b**). Systolic view; (**c**). Early T1 acquisition; (**d**). T2 acquisition; (**e**,**f**). LGE sequences: mid-myocardial contrast uptake with non-ischemic pattern at the interventricular septum and right ventricular insertion junction; (**g**,**h**). Right atrium diverticulum (red arrow).

Additionally, MRI identified a well-defined, intrapericardial pouch measuring approximately 20 cm^2^, located anterior to the right atrium, with a 15 mm communication to the atrial cavity, suggestive of a right atrial diverticulum (Figure 3).

**Figure 4:**
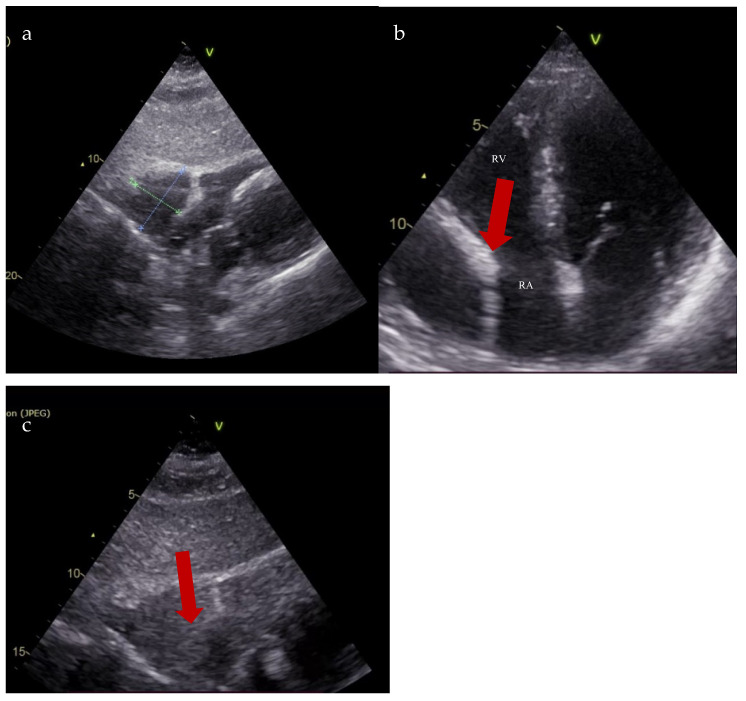
Transthoracic echocardiography. (**a**). Subcostal view: right atrial diverticulum 36 mm × 49 mm; (**b**). 4-chamber apical section: right atrial diverticulum with systolic collapse of the right atrium (red arrow); (**c**). Saline contrast test—subcostal section: communication between right atrial diverticulum and right atrium (red arrow) (RA—right atrium; RV—right ventricle).

Follow-up transthoracic echocardiography revealed an enlargement of the previously noted structure, now measuring 36 × 49 mm, producing the systolic collapse of the right atrium. A saline contrast study confirmed the communication between the right atrium and the pericardial space (Figure 4).

**Figure 5:**
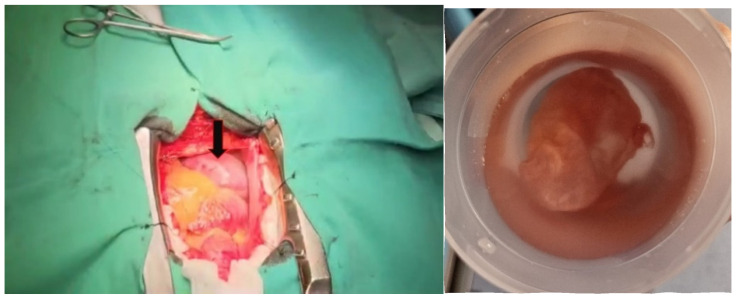
Intraoperative visualization of the right atrium diverticulum (black arrow).

Due to the increased size of the diverticulum and the associated high risk of rupture, the patient was referred to the Cardiovascular Surgery Department, where a successful resection of the lesion was performed (Figure 5). The histopathologic examination confirmed a bistratified wall consisting of a thin endothelium and fibrous tissue with peripheral collagenization, suggestive of a diverticulum. The patient followed the same treatment as before with doses adjusted to clinical and paraclinical data (carvedilol 3.125 mg twice a day, sacubitril/valsartan 24/26 mg twice a day, dapagliflozin 10 mg once a day, spironolactone 50 mg once a day, and rosuvastatin/ezetimibe 40/10 mg once a day).

The patient was followed up at one week, one month, six months, and then annually. The patient remained asymptomatic, and transthoracic echocardiographic reevaluation demonstrated the resolution of “pericardial effusion” and improved systolic function, with a left ventricular ejection fraction of 52%.

## 3. Discussion

The clinical presentation of the right atrial diverticulum is variable and may include dyspnea [12,18], chest pain [13,17], fatigue, or palpitations [6]. The symptoms are mainly caused by the mechanical compression of the adjacent cardiac cavities.

In our case, dyspnea was caused by the severe left ventricular systolic dysfunction, considering the marked improvement in terms of the enhancement of systolic function after optimal therapy.

Palpitations are frequently the clinical expression of supraventricular arrhythmias (atrial extrasystoles, supraventricular tachycardias, atrial fibrillation, atrial flutter), and are often resistant to antiarrhythmic drugs. The arrhythmias result from circus movement reentry (atrial re-entrant tachycardias) or from direct stimulation of the cardiac surface [6,19]. It has been reported that some cases of small right atrial diverticulum were discovered on cardiac mapping for ablation [6,10,11]. In such cases, the 3D electroanatomical reconstruction system coupled with right atrial computed tomography angiography is mandatory for the definitive confirmation of arrhythmia origin [20].

Most frequently, patients are asymptomatic and the discovery of a right atrium diverticulum by cardiac imaging methods such as transthoracic and transesophageal echocardiography, cardiac computed tomography, and cardiac MRI [14,16,21] is incidental. The discovery of the right atrium diverticulum when complications such as intracavitary thrombus [21], rupture, or sudden death [7,14] have occurred has also been described. Sudden cardiac death was described in 6% of congenital malformations of the right atrium [1].

In the absence of supraventricular arrhythmias, an electrocardiogram is nonspecific, although several authors have attempted to characterize discrete end-phase changes caused by a change in the cardiac axis [13]. If arrhythmias are present, a surface electrocardiogram is a valuable resource in the initial evaluation [6,10,11].

Transthoracic echocardiography is a valuable imagistic method for the diagnosis of right atrial diverticulum, but it must be differentiated from a localized pericardial effusion, or a pericardial cyst [1].

Right atrial diverticulum is an extremely rare congenital malformation and few cases have been reported in the literature. Over the years, there has been an overlap between the terms right atrial aneurysm [9,22] and right atrial diverticulum [5,7], which actually are two distinct malformations [1]. Right atrial aneurysm, first described by Bailey et al. in 1955 [2], is more commonly encountered, and consists of right atrial dilatation with tricuspid annulus involvement and secondary tricuspid regurgitation [1]. The cause of congenital right atrial aneurysms is muscular wall dysplasia, with a muscular rim alongside the crista terminal or the superior vena cava. Histopathologically, it consists of a decreased muscular layer, endocardial fibrosis, and a fatty degeneration wall [23]. It may be isolated or may be associated with other cardiac abnormalities such as an atrial septal defect [23,24] or even a left atrial aneurysm [24].

The right atrial diverticulum is a pouch-like structure originating from the auricle or the right atrial wall, which communicates with the right atrium [1]. The proposed embryologic origin differs depending on the localization of the diverticulum. The diverticula placed posterior to the wall of the right atrium often involve the coronary sinus and are caused by the incomplete regression of Cuvier’s ducts. During fetal development, the right duct of Cuvier becomes the inferior vena cava, while the left duct regresses after four weeks of pregnancy and becomes the coronary sinus or superior vena cava. The diverticula localized anterior to the right atrium are thought to derive from the auricle [5,7].

The etiology of these diverticula is unclear. Protein or collagen type III deficiency (in Ehlers Danlos syndrome type IV) is supposed to be involved in decreased tissue strength [17]. Some cases with a right atrium diverticulum wall consisting only of the outer layer have been described. The wall was very thin with a high risk of rupture [14]. In our case, the diverticulum wall consisted of an outer fibrous layer and an inner endothelial layer, as is usually described in the literature [4,12,14,16,18].

There are no standardized treatment guidelines for right atrial diverticulum. Therapeutic approaches must be individualized. Surgical correction is recommended for large or complicated diverticula [12,18], while a conservative strategy and follow-up are suitable in asymptomatic patients [13]. In most cases reported in the literature, surgery with cardiopulmonary bypass has been chosen due to its superior safety profile regarding the risk of right atrial rupture [4,12]. The communication between the right atrial diverticulum and the right atrium is closed by a continuous suture [14] or an autologous pericardial patch [4].

If the arrhythmia coexists, radiofrequency ablation is often performed after surgical resection. For small diverticula, the procedure can be performed before the surgical correction [25]. The techniques of the arrhythmia-maintaining circuit ablation are often challenging because of the varied geometry of the right atrial diverticulum [6,10,21,25]. In the case of a small right atrial diverticulum complicated with reintrant atrial tachycardia and identified on an electroanatomic 3D reconstruction model, ablation was successfully performed at the junction between the right atrium and the diverticulum. The fibrous connective tissue between the right atrium and the diverticulum is usually the substrate of arrhythmogenesis [20]. If conservative treatment and follow-up is chosen, an anticoagulant is associated with the prevention of thrombus formation [1,4,10,25].

## 4. Conclusions

The particularity of the case lies is the incidental identification of a rare congenital cardiac malformation in an adult patient. The patient presented to the Cardiology Department for persistent dyspnea post COVID-19 infection. Initial work-up showed severe systolic LV dysfunction and a localized pericardial effusion on echocardiographical evaluation. Cardiac MRI was performed to confirm viral myocarditis as the etiology of heart failure, and surprisingly revealed that “the localized pericardial effusion” was in fact a right atrial diverticulum, a well-defined intrapericardial anatomical structure communicating with the right atrium. The increased size and the related risk of rupture required surgical resection, which was successfully performed.

## Figures and Tables

**Table 1:** Laboratory parameters.

Blood count and inflammation markers	Hemoglobin 15 g/dL Hematocrit 45.9% Red blood cells 5,460,000/mm^3^ Platelets 251,000/mm^3^ White blood cells 6180/mm^3^ Protein C reactive 0.3 mg/dL Fibrinogen 374 mg/dL
Liver function	Aspartate aminotransferase 23 U/L Alanine aminotransferase 27 U/L Gamma-glutamyl transferase 18 U/L
Renal function	Urea 54 mg/dL Creatinine 0.6 mg/dL GFR_e_ 101 mL/min/1.73 m^2^ Na^+^ 143 mmol/L K^+^ 4.8 mmol/L
Cardiac biomarkers	NT-proBNP 2869 pg/mL Creatine kinase MB izoenzyme 20 U/L High-sensitivity cardiac troponine test negative
Cardiovascular risk profile	Total cholesterol 219 mg/dL LDL-cholesterol 170 mg/dL HDL-cholesterol 44 mg/dL Non-HDL cholesterol 175 mg/dL Triglyceride 118 mg/dL Blood glucose 89 mg/dL Uric acid 4.8 mg/dL

**Table 2:** Echocardiographic parameters.

LVDd	49 mm	RVD basal	25 mm
IVST	8 mm	TAPSE	20 mm
PWT	8 mm	RV-RA gradient	42 mmHg
E/A	1.2	PAP	47 mmHg
mean E/E’	15		
LVEF	20%		
LA area	22 cm/m^2^	RA area	20 cm/m^2^
LA volume	33 mL/m^2^	RA volume	25 mL/m^2^

LVDd, left ventricular end-diastolic diameter; IVST, thickness of the interventricular septum; PAP, pulmonary artery systolic pressure; PWT, thickness of the LV posterior wall; TAPSE, tricuspid annular plane systolic excursion; RVD basal, basal right ventricle diameter.

## Data Availability

The data presented in this study are available from the corresponding author upon request. The data are not publicly available due to the confidentiality of personal data.

## Abbreviations

The following abbreviations are used in this manuscript:

COVID 19	Coronavirus disease 2019
LGE	late post-contrast sequences
MRI	magnetic resonance imaging
